# Dual-mode optical projection mapping system: integration of laser speckle contrast and subcutaneous vein imaging

**DOI:** 10.1007/s13534-024-00400-4

**Published:** 2024-06-07

**Authors:** Insun Yeum, Donghwan Ko, Gyujin Lee, Hoik Seok, Byungjo Jung

**Affiliations:** Department of Biomedical Engineering, Yonsei University, Wonju-Si, Republic of Korea

**Keywords:** Laser speckle contrast image, Subcutaneous vein image, Projection mapping, Dual-mode optical imaging

## Abstract

Dual-mode optical imaging can simultaneously provide morphological and functional information. Furthermore, it can be integrated with projection mapping method to directly observe the images in the region of interest. This study was aimed to develop a dual-mode optical projection mapping system (DOPMS) that obtains laser speckle contrast image (LSCI) and subcutaneous vein image (SVI) and projects onto the region of interest, minimizing the spatial misalignment between the regions captured by the camera and projected by a projector. In in vitro and in vivo studies, LSCI and SVI were obtained and projected under single-mode illumination, where either the laser or light-emitting diode (LED) was activated, and under dual-mode illumination, where the laser and LED were activated simultaneously. In addition, fusion image (FI) of LSCI and SVI was implemented to selectively observe blood perfusion in the vein. DOPMS successfully obtained LSCI, SVI, and FI and projected them onto the identical region of interest, minimizing spatial misalignment. Single-mode illumination resulted in relatively clearer and noise-free images. Dual-mode illumination introduced speckle noise to SVI and FI but enabled real-time imaging by simultaneously employing LSCI, SVI, and FI. FI may be more effective for quasi-static evaluations before and after treatment under single-mode illumination and for real-time evaluation during treatment under dual-mode illumination owing to its faster image processing, albeit with a potential tradeoff in image quality.

## Introduction

Laser speckle contrast imaging is a noninvasive technique that utilizes the contrast variations in laser speckle patterns caused by scattering moving particles, such as red blood cells in vasculature, to monitor blood flow and perfusion [1, 2]. It has been widely used in various medical diagnostic applications, such as monitoring microcirculation, observing blood flow during surgery, and evaluating blood perfusion following transplantation [3–6]. Laser speckle contrast image (LSCI) can effectively identify vasculature when it is clearly observable and exposed, such as in cerebral vasculature [7–11]. However, conventional LSCI was displayed on a monitor screen and presented relatively less vascular morphology when the vascular is not highly visible [12–14]. Therefore, accurately identifying the blood perfusion on the lesion can be challenging when the vasculature is not highly visible. Even when the vasculature is highly visible, a spatial misalignment between the vasculature lesion and monitor screen could occur, posing potential inaccuracy in analysis [15]. To partially solve this issue, previous studies have incorporated the LSCI with a white light image on a monitor screen or used a projector to overlay the LSCI image on a surgical microscope [16, 17].

Subcutaneous vein image (SVI) uses near-infrared light to selectively enhance vein morphology by maximizing the contrast difference between the vein and surrounding tissue [18–20]. SVI was combined with a projection mapping method, which projected a near-infrared vein image in real-time onto an identical body location to directly observe the vein in the region of interest [21]. Although SVI has been successfully employed for subcutaneous vein detection in intravenous applications and evaluating various vascular disorders, it provides only vein morphology [22, 23].

Projection mapping uses a projector to display images or videos on two-dimensional or three-dimensional surfaces, such as the human body, complex natural landscapes, or industrial environments. Commercial projection mapping software interact with a projector and adjust the projection region to fit the specific surface, allowing for a seamless and accurate display [24]. Projection mapping was used in conjunction with magnetic resonance imaging to project images of the tumor onto the breast, allowing for more precise and accurate surgical removal of the tumor while preserving as much healthy tissue as possible [25]. Indocyanine green fluorescence imaging was combined with projection mapping to project images of cancerous tissue onto the body, making it easier for surgeons to identify and remove the cancerous tissue [26]. Recently, LSCI was combined with projection mapping to identify and track the blood perfusion variation in the human body [14]. In medical education, projection mapping may be used to visualize the human anatomy on a live human body model, allowing interactive study of the human anatomy in a more engaging and informative way [27, 28].

This study developed a dual-mode optical projection mapping system (DOPMS) that obtains LSCI, SVI, and a fusion image (FI) and projects onto the imaging region of interest (IROI), minimizing the spatial misalignment between the IROI captured by the camera and the region of interest projected by a projector. The FI was designed to selectively highlight blood perfusion in vasculature, combining functional information about blood perfusion from LSCI with morphological information about the vasculature from SVI. The FI aims to improve the accuracy and efficacy in identifying and evaluating vascular function and morphology in medical diagnostics. A graphical user interface software was developed to process the LSCI and SVI, as well as the FI. In vitro and in vivo evaluations of the DOPMS were performed to investigate its efficacy and potential clinical applications.

## Materials and methods

### Development of DOPMS

**Fig. 1:**
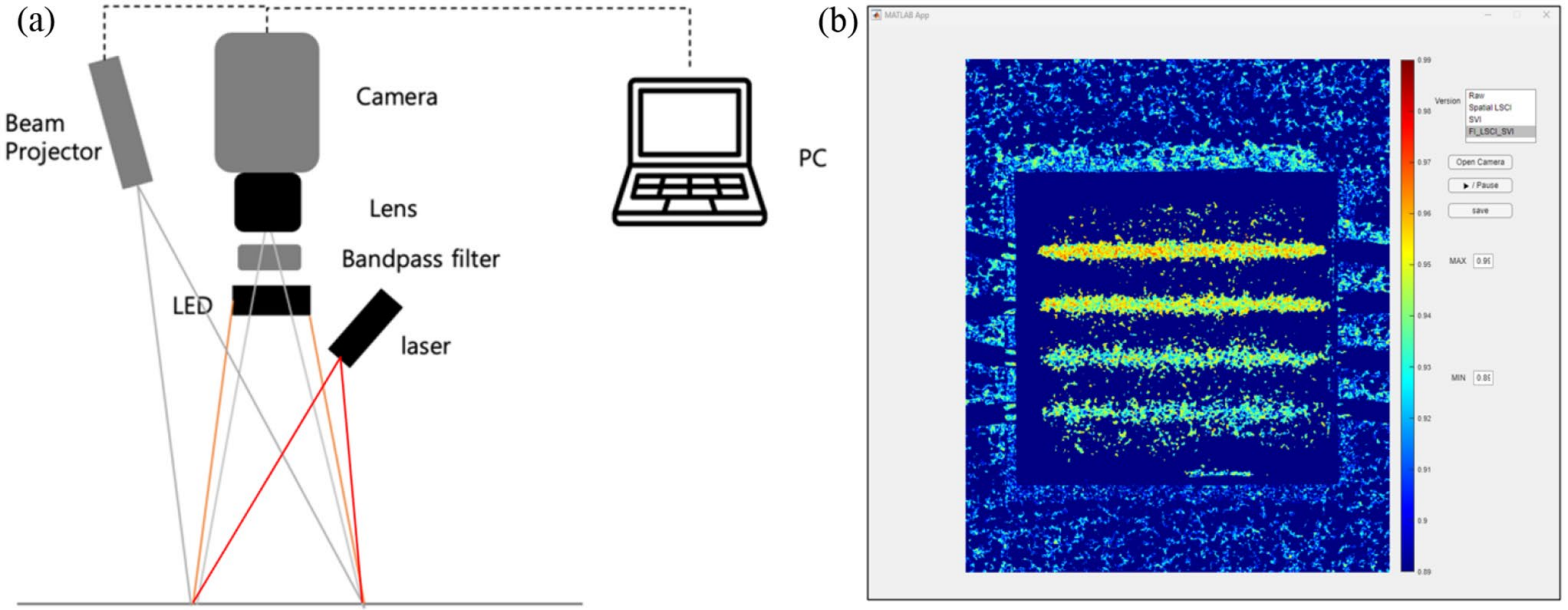
(**a**) Schematic diagram of dual-mode optical projection mapping system and (**b**) graphical user interface software

Figure 1(a) shows the schematic diagram of DOPMS that consists of a monochrome camera (CS135MUN, Thorlabs, USA), 16 mm fixed focal length lens (67–714, Edmund Optics, USA), laser projector (MP-CL1, Sony, Japan), long-pass filter with a cut-on wavelength of 750 nm (84–761, Edmund Optics, USA), 785 nm laser diode (L785P090, Thorlabs, USA), diffuser with a diffusing angle of 60° (35–869, Edmund Optics, USA), and 850 nm light-emitting diode (LED) (WL-SITW IR850nm, Wurth Electronic, Germany). The DOPMS has a 26 cm working distance and 7.5 cm $\times$ 9.5 cm region of interest. The laser projector was placed close to the camera at a beveled angle.

Figure 1(b) shows the graphical user interface software developed using MATLAB (MATLAB, MathWorks, USA) for image processing and display of the raw image, LSCI and SVI, and the FI for selective monitoring of blood perfusion in the vein. The software development kit provided by the camera manufacturer was used to enhance the image processing speed. The DOPMS was operated using a laptop computer equipped with a high-performance CPU (i7-11800 H 32GB, Intel, USA) and GPU (GeForce FTX 3060 Laptop GPU, NVIDIA, USA). The image processing speed was determined in frames per second (fps) by averaging the processing time across 50 iterations for each imaging mode.

### Image processing of FI

**Fig. 2:**
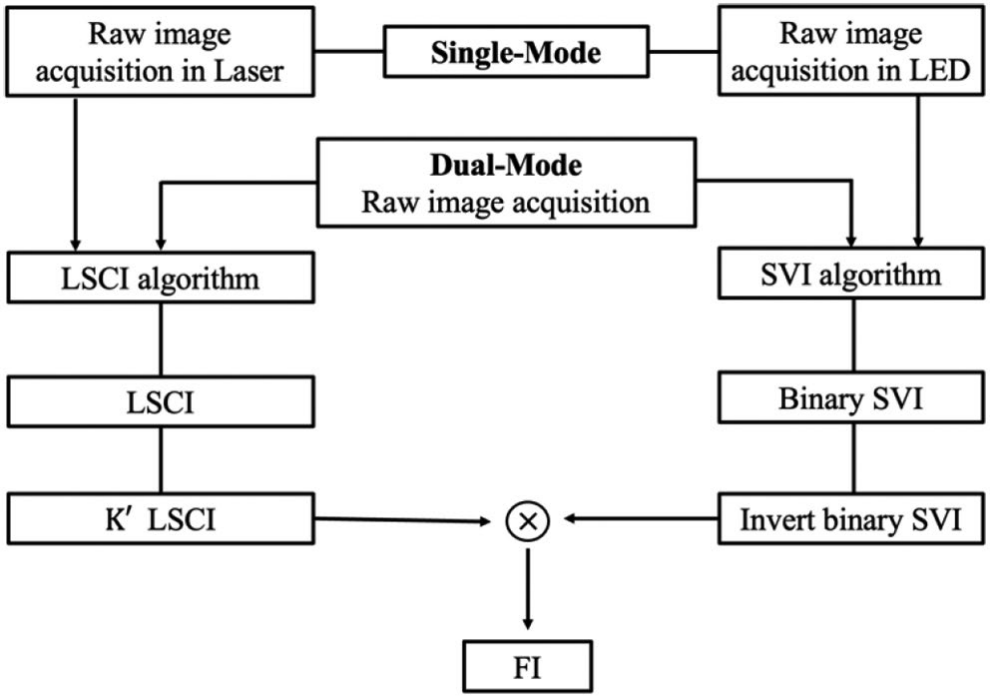
Image processing procedure to obtain LSCI, SVI, and FI under single-mode and dual-mode illumination

Figure 2 shows the image processing procedure to obtain FI under single-mode and dual-mode illumination. Under dual-mode illumination, both laser and LED are illuminated simultaneously to obtain a raw image. The raw image was converted into double format because a previous study demonstrated that using the double format in LSCI effectively eliminated rounding errors and provided reasonable results [29]. The raw speckle image is first processed using the LSCI algorithm. The speckle contrast of K is calculated from the LSCIs and then, reversed into K’ to intuitively provide flow velocity variation as follows:

1 $$speckle \,contrast \left(K\right) = \sigma /\mu\, \text{a}\text{n}\text{d}\, K^{\prime } = 1 - K$$

where σ is the standard deviation of the pixel intensity values of a 5 by 5 moving window in the raw speckle image and µ is the mean of the pixel intensity values within the same window. The closer K’ is to 0, the lower the flow velocity, and the closer K’ is to 1, the faster the flow velocity.

The identical raw image was further processed using the SVI algorithm that generates a binary image, where the pixel values in the vasculature become 0. These values are then inverted, setting vasculature pixels to 1 and non-vasculature pixels to 0. The inverted SVI is then multiplied by the previously processed LSCI. As a result, the K’ values of LSCI in the vasculature remain unchanged but become 0 in the non-vasculature, presenting blood perfusion selectively in vasculature. Under single-mode illumination, raw images are independently obtained under laser illumination for LSCI and LED illumination for SVI. Subsequently, an identical image processing procedure, as applied in dual-mode illumination, is used to obtain FI.

### Evaluation of light distribution of light sources

The DOPMS can be operated under either single-mode illumination, which independently activates either the laser or LED, or dual-mode illumination, which simultaneously activates both the laser and LED. The two-dimensional light distribution images under single- and dual-mode illumination were obtained using a standard diffuse reflectance target (SRT-99-100, Labsphere Inc., USA) with 99% reflectance. The light distribution was quantitatively evaluated by calculating the coefficient of variation at the full width at half maximum of the intensity profile obtained by performing a column average of the light distribution image. For the calculation of the coefficient of variation, the 256 grayscale images were transformed into a double format, which rescaled the pixel values within a range of 0–1.

### Evaluation of spatial alignment in projection mapping

A resolution test target (Yoshihiko Takinami, USAF 1951 target) was placed at the working distance to simultaneously evaluate the spatial alignment between the IROI and projection region of interest (PROI), and the projection mapping resolution of the DOPMS. Additional evaluations of spatial alignment were performed by attaching markers to an in vivo dorsal hand and in vitro vascular optical tissue phantom. The optical tissue phantom was fabricated using room-temperature-vulcanizing silicone with optical tissue properties (µ_a_ of 0.12 and µ_s_^′^ of 2.3) at 785 nm, resulting in error of 6.19% of µ_a_ and 5.99% of µ_s_^′^ in the measurement using invers adding doubling program. Silicone tubing (Si012, DAIHAN Pure Silicone Robber Tubing, SciLab, Korea) with inner diameter of 2 mm and our diameter of 3 mm was placed at four depths (0.9, 1.1, 1.3, and 1.5 mm) to simulate the blood vessels at various tissue depths.

To evaluate spatial alignment, near-infrared images were obtained with the DOPMS from the IROI of the samples, and their screen was projected and manually aligned by adjusting the location of the software screen to align the locations of the markers between the PROI and IROI. Although the projector was placed with a beveled angle that may cause projection mapping distortion, it was easily corrected by applying the “Keystone” correction function of the projector [30]. White light images of the projection mapping in the IROI were obtained using an iPhone camera placed close to the camera of the DOPMS. The error of spatial alignment was obtained by calculating the offset pixel numbers between markers in the IROI and the PROI as follows:

2 $$\frac{\text{A}}{\text{B}+\text{C}}\times 100$$

where, A denotes the total number of offset pixels between markers in the IROI and PROI, while B and C denote the number of pixels of markers in the IROI and PROI, respectively. Markers were selected in both IROI and PROI to count these pixels. The white light image was then converted into a binary image, in which offset areas were marked with 1 and all other areas with 0. Subsequently, the pixels corresponding to the offset were counted.

### Evaluation of light interference in imaging

LSCI, SVI, and FI were obtained under single-mode and dual-mode illumination to investigate light interference in imaging by analyzing the flow velocity and tubing morphology in the vascular optical tissue phantom. A 1% milk solution was circulated through the vascular optical tissue phantom using a peristaltic pump (PP-150D, PLTECH, Korea) equipped with LS/16 hose (I.D: 3.1 mm/O.D:6.3 mm) at a 200 revolutions per minute (RPM). LSCI and SVI were independently obtained activating laser and LED under single-mode illumination, respectively, and simultaneously obtained under dual-mode illumination. FI was obtained under single- and dual-mode illumination by combining LSCI and SVI to selectively evaluate the flow velocity in the tubing region extracted through SVI. Irradiation in the IROI was optimized for each illumination mode by adjusting aperture size to avoid image saturation.

### Projection mapping of in vitro and in vivo images

A 1% milk solution was circulated through the vascular optical tissue phantom using a peristaltic pump (PP-150D; PLTECH, Seoul, Korea) at 0, 100 (80 ml/min), and 200 (160 ml/min) RPM. LSCI, SVI, and FI were obtained under single-mode and dual-mode illumination and projected onto the IROI to evaluate the projection mapping depending on the illumination mode.

A sphygmomanometer cuff was applied to the right upper arm of a volunteer to evaluate variations in blood perfusion. The blood pressure cuff was inflated to approximately 200 mmHg, and the occlusion of the blood flow was maintained for about 2 min. LSCI, SVI, and FI were obtained from the right dorsal hand under single- and dual-mode illumination and projected onto the IROI at three different blood pressure states: normal without occlusion, occlusion by tightening the cuff, and reperfusion by releasing the cuff. The in vivo experiment was approved by the Bioethics Committee of Yonsei University (1041849-202111-BM-192-01).

## Results

### Evaluation of light distribution of light sources

**Fig. 3:**
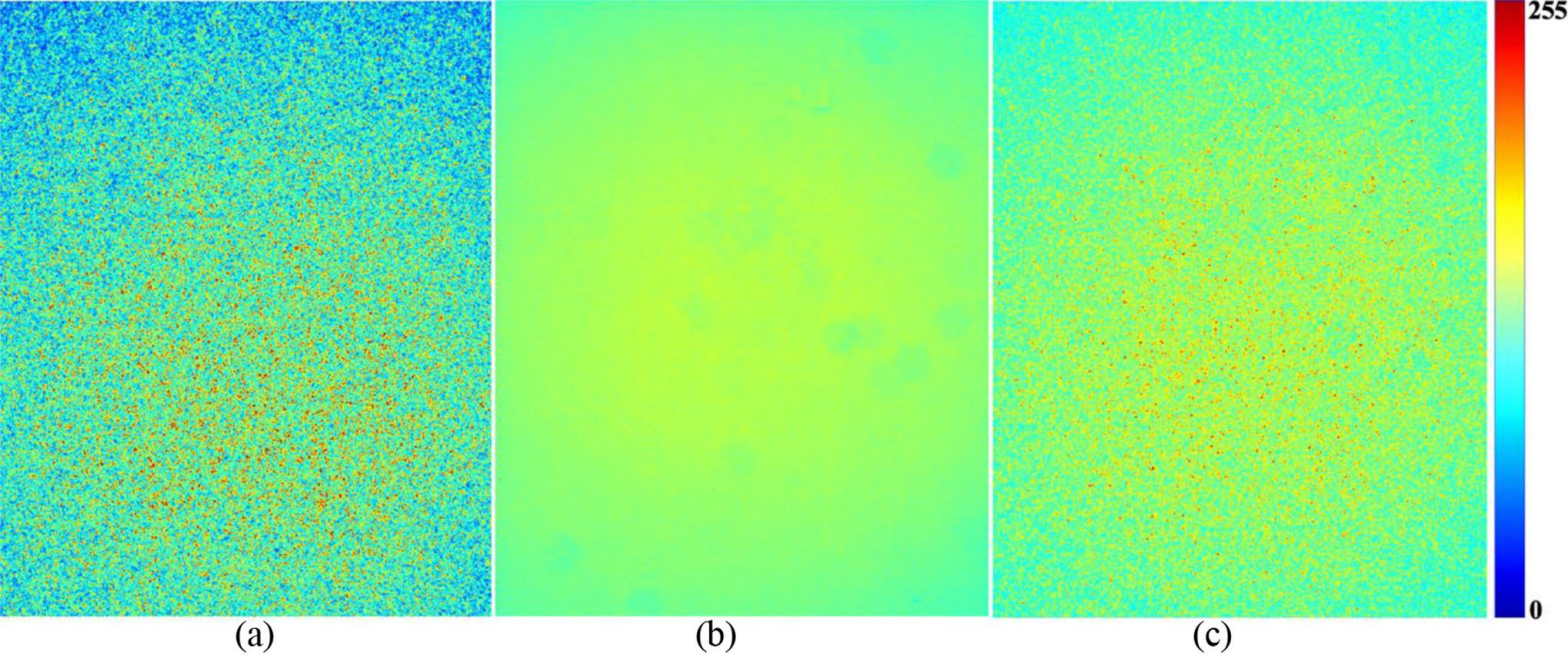
Two-dimensional light distribution images of (**a**) laser, (**b**) LED, and (**c**) dual-mode illumination. The color bar denotes the range of brightness in the light distribution

Figure 3 shows the two-dimensional light distribution images of the (a) laser, (b) LED, and (c) dual-mode illumination. It shows a quasi-flat even-light distribution, presenting a low coefficient of variation of 5.40, 2.31, and 3.27% for laser, LED, and dual-mode illumination, respectively. LED illumination resulted in the lowest coefficient of variation, with a relatively lower fluctuation of the high-frequency intensity than the others. Laser illumination resulted in a relatively high fluctuation in the high-frequency intensity owing to speckle noise, which was not observed in the LED illumination. Dual-mode illumination resulted in relatively less fluctuation of the high-frequency intensity than the laser illumination. It may be achieved by optimally adjusting the aperture size to avoid image saturation and to balance the intensity levels of the laser and LED.

### Evaluation of spatial alignment in projection mapping

**Fig. 4:**
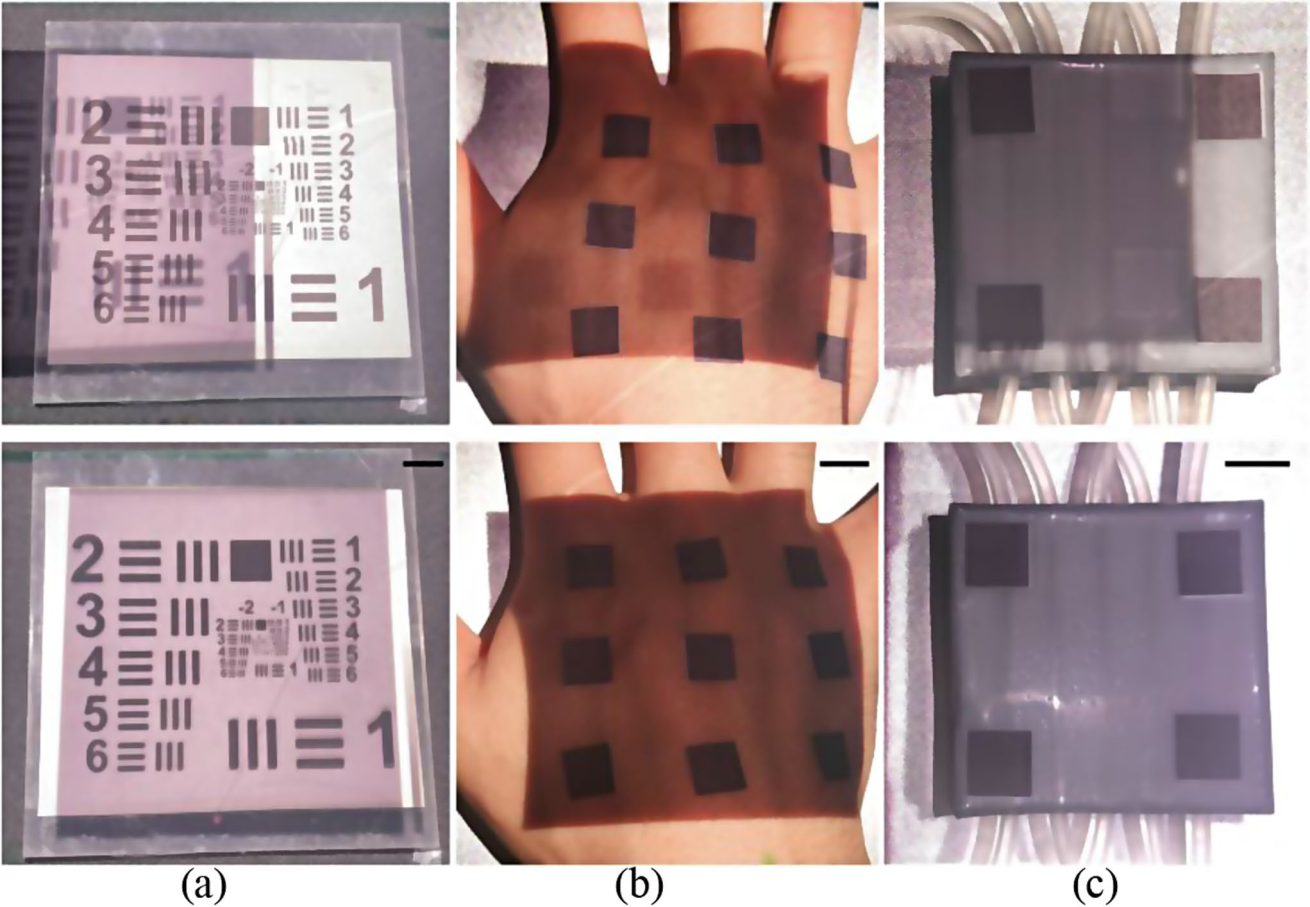
Spatial alignment procedure between the IROI and PROI on (**a**) a resolution test target board, (**b**) in vivo dorsal hand, and (**c**) in vitro vascular optical tissue phantom. The upper- and lower-row images show adjustment procedure and completion of the spatial alignment, respectively. The black solid bars in lower images indicate scale bars of 10.5 mm.

Figure 4(a) to (c) show the spatial alignment procedure on a resolution test target, in vivo dorsal hand, and in vitro vascular optical tissue phantom, respectively. The upper-row images show the projection and adjustment procedure of the near-infrared images obtained in IROI. The bottom-row images show the completion of the spatial alignment procedure, indicating that the projection mapping was completely aligned with the samples. The errors of spatial alignment were 3.50 and 1.68% in Fig. 4(b) and (c), respectively. A relatively higher error was observed in Fig. 4(b), likely due to the curvature of the dorsal hand. Once spatial alignment was completely established with projection mapping of the near-infrared images, the DOPMS maintained the location of the software screen without any additional spatial alignment procedure, even in the absence of markers in both in vitro and in vivo studies.

### Evaluation of light interference in imaging

**Fig. 5:**
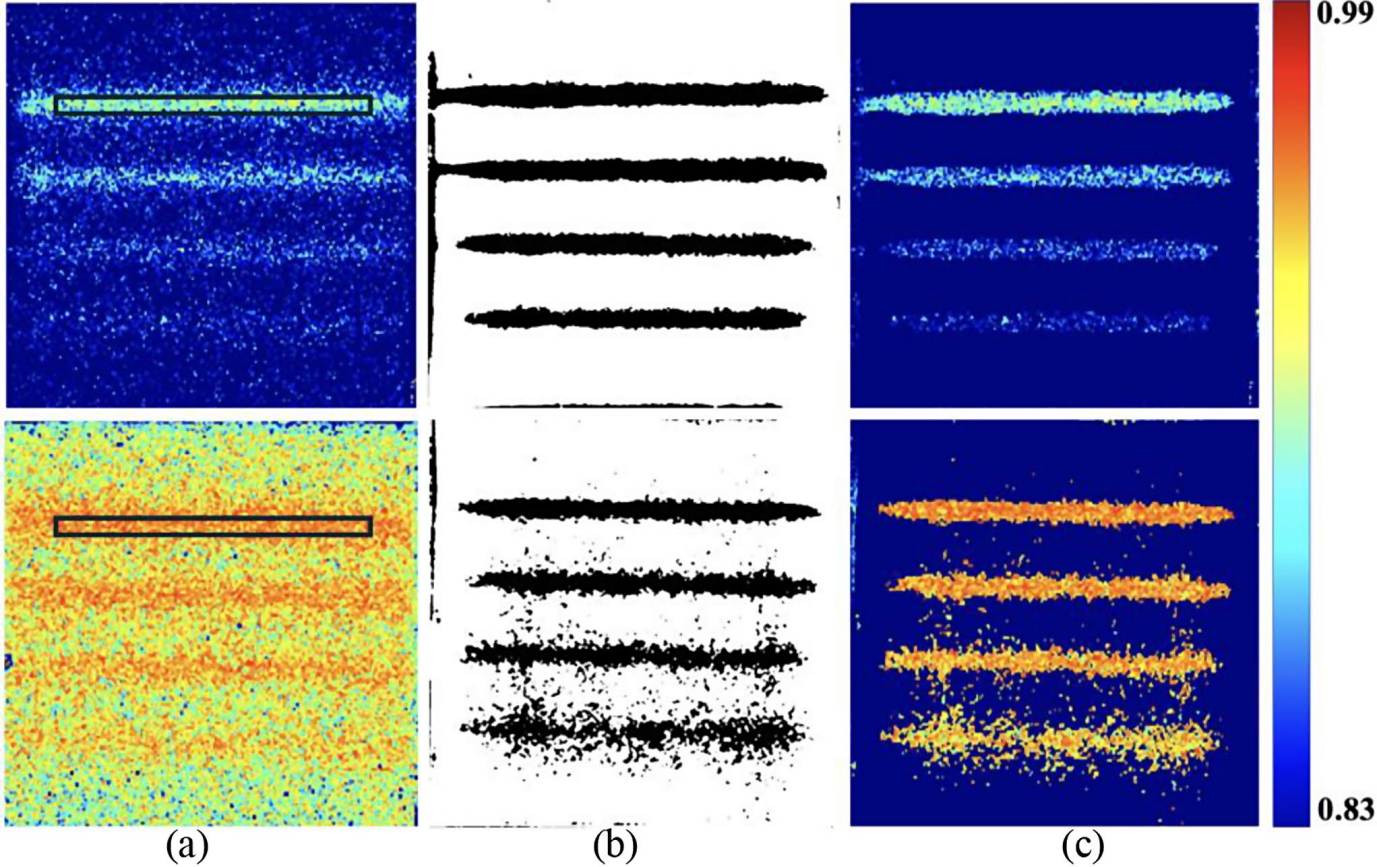
(**a**) LSCI, (**b**) SVI, and (**c**) FI in single-mode (upper-row images) and dual-mode (bottom-row images) illumination at 200 RPM. The images were obtained from vascular optical tissue phantom. The color bar denotes the range of K’ in the first and third column images

Figure 5(a) to 5(c) show LSCI, SVI, and FI under single-mode (upper-row images) and dual-mode (bottom-row images) illumination, respectively. The image processing speeds were 38.4, 18.08, and 11.36 fps for the LSCI, SVI, and FI, respectively. Average K’ values were calculated at 0.9 mm tubing depth region (marked as black solid rectangular bar) and resulted in 0.90 for single-mode illumination and 0.95 for dual-mode illumination, both at 200 RPM. LSCI under dual-mode illumination resulted in higher flow velocities compared to single-mode illumination at the same RPM. Furthermore, deeper tubing resulted in relatively lower K’ values than shallow tubing at the same RPM. Shallow tubing presented a relatively higher flow velocity, lower perfusion, and better tubing morphology than deep tubing. The SVI presented a clearer tubing morphology under single-mode illumination across four different depths, whereas the image under dual-mode illumination presented a quasi-distinct tubing morphology with speckle noise. The FI presented greater selectivity in the flow velocity within the tubing under single-mode illumination, whereas dual-mode illumination introduced higher speckle noise around the tubing.

### Projection mapping of in vitro image

**Fig. 6:**
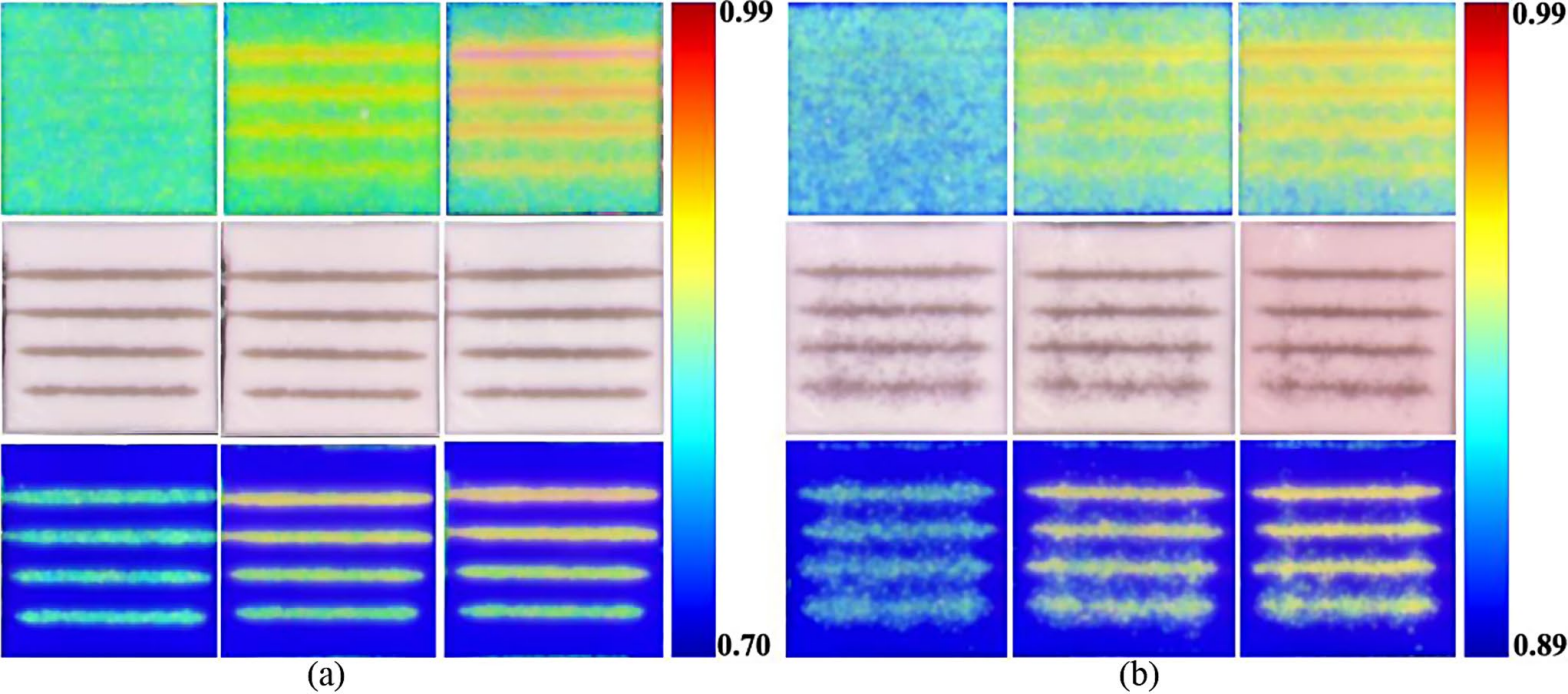
Projection mapping of LSCI (upper-row images), SVI (middle-row images), and FI (bottom-row images) under (**a**) single-mode and (**b**) dual-mode illumination from vascular optical tissue phantom. The color bars denote the range of K’ in the upper-row and bottom-row images

Figure 6 shows the projection mapping of the LSCI (upper-row images), SVI (middle-row images), and FI (bottom-row images) under (a) single-mode and (b) dual-mode illumination at 0 (left-column images), 100 (middle-column images), and 200 RPM (right-column images). The range of the color bar was adjusted to enhance the contrast of flow variation depending on the illumination mode. Average K’ was calculated at 0.9 mm tubing depth region. In single-mode illumination, the average K’ values were 0.78 at 0 RPM, 0.87 at 100 RPM, and 0.90 at 200 RPM. In dual-mode illumination, the corresponding values were 0.91, 0.93, and 0.95, respectively. SVI under single-mode illumination resulted in a clearer tubing morphology across all RPMs than that under dual-mode illumination, which presented speckle noise, particularly around the deeper tubing. The entire tubing morphology was detected at shallow depth, whereas neither end of the tubing morphology was detected at relatively deeper depths. Under single-mode illumination, FI presented the flow velocity selectively within the tubing obtained through SVI, whereas speckle noise around the tubing was observed under dual-mode illumination. This may have clinical applications by providing more selective blood perfusion information within the vascular.

### Projection mapping of in vivo image

**Fig. 7:**
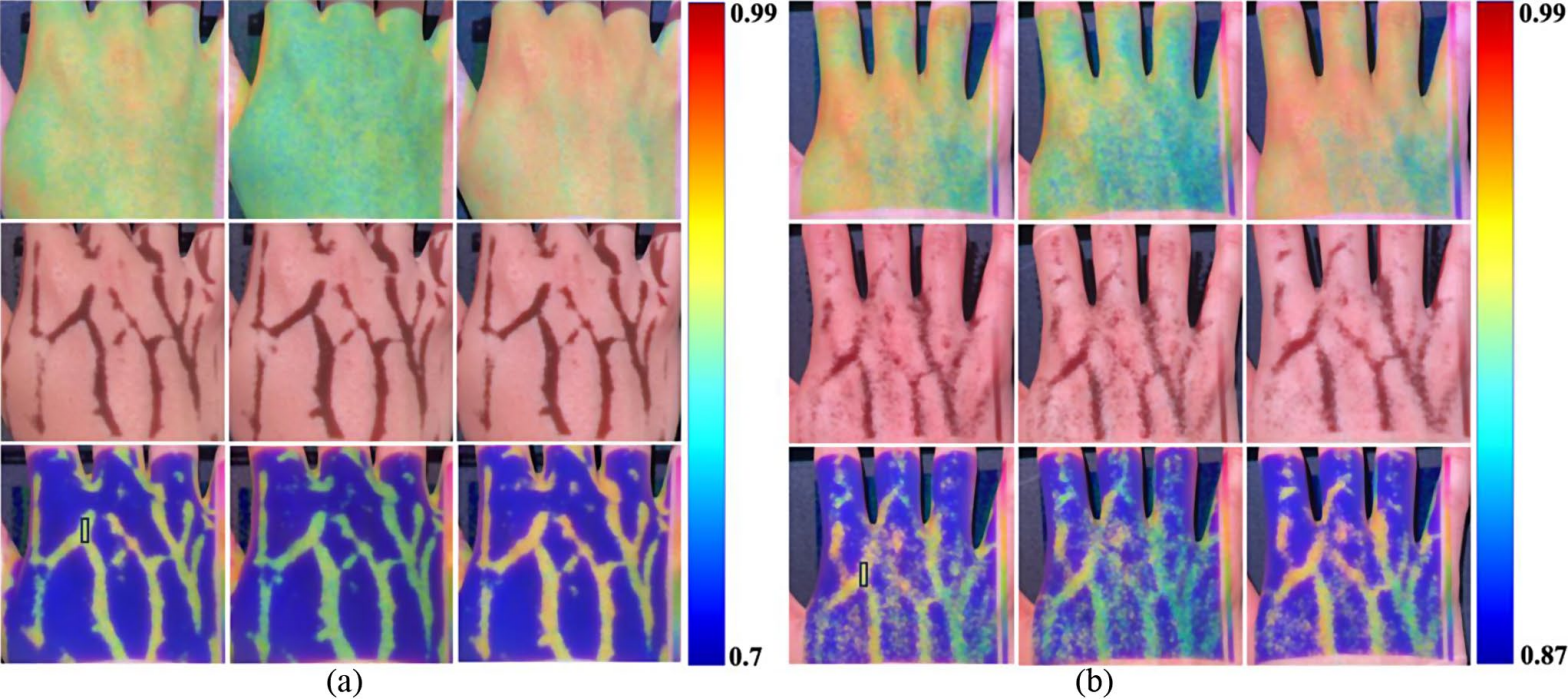
Projection mapping of LSCI (upper-row images), SVI (middle-row images), and FI (bottom-row images) obtained under (**a**) single-mode and (**b**) dual-mode illumination without occlusion (left-column images), with occlusion by tightening the cuff (middle-column images), and with reperfusion by releasing the cuff (right-column images). The color bars denote the range of K’ in the upper-row and bottom-row images

Figure 7 shows the projection mapping of the LSCI (upper-row images), SVI (middle-row images), and FI (bottom-row images) on the right dorsal hand under (a) single-mode and (b) dual-mode illumination. The range of the color bar was adjusted to enhance the contrast of flow variation depending on the illumination mode. Although the LSCI presented blood perfusion variation as a function of blood pressure, Fig. 7(a) and (b) show different blood perfusion velocities for three different blood pressures due to different measurement time points, although attempts were made to obtain the LSCI at similar time points, and different laser intensities depending on the illumination mode. In the solid rectangular bars shown in Fig. 7, the single-mode (dual-mode) illumination resulted in average K’ values of 0.86 (0.92) in the normal state, 0.83 (0.90) during occlusion, and 0.89 (0.93) upon release. In all cases, the average K’ values decreased during occlusion compared to the normal state, and the highest average K’ values were observed in the release state. Unlike in vitro study, vein morphology was not clearly observed in LSCI. LSCI provides a perfusion image when vascular is not clearly exposed, although the visibility of the vascular may vary depending on the subject. SVI presented a clearer vein morphology under single-mode illumination than under dual-mode illumination, which presented speckle noise around the vein morphology, leading to reduced vein detectability. FI selectively presented clearer blood perfusion within the vein under single-mode illumination than under dual-mode illumination.

## Discussion

Projection mapping has various potential applications in medical diagnoses and surgical procedures. It can be used for the realistic simulation of medical procedures in a safely controlled environment and during actual surgical procedures to provide intuitive real-time information and guidance to the surgeon. However, projection mapping is still in the early stages of development for medical applications, and further studies are necessary to effectively integrate it into medical procedures and enhance its efficiency. Spatial alignment in projection mapping is crucial in clinical application to avoid lesion mismatches between the IROI and PROI. Spatial alignment was successfully verified through both in vitro and in vivo studies, and established across various samples, demonstrating its clinical feasibility. In future studies, an advanced projection mapping algorithm for spatial alignment should be developed and included in the graphical user interface software by considering commercial software.

LSCI provides information about the dynamics of blood flow and perfusion [31], while SVI provides morphological information about the vasculature [32]. Typically, LSCI can visualize blood perfusion to a depth of about 1–2 mm from the skin surface [33]. The depth of human veins in the periphery usually ranges from 1.4 mm to 2.59 mm, varying based on individual factors like body fat percentage [34, 35]. Although the exact depth of veins wasn’t known in the study subject, successful SVI was achieved, indicating that the LED reached the vein depth under dual-mode illumination.

LSCI can effectively identify vasculature when it is clearly observable and exposed, such as in cerebral vasculature. However, its effectiveness diminishes in less visible areas, like the dorsal hand. Various previous studies involve images of the cerebral cortex of mice. If thin skull windows or surgically non-invasive optical clearing windows are used, it is possible to monitor vasculature resolution using conventional LSCI. However, unless in such cases, light must pass through the superficial tissue layers before reaching the deep vasculature layer, and the static speckle intensity of the superficial tissue layer is much greater than the dynamic speckle signal of the deeper vasculature. This may result in a lower signal-to-background ratio, making it difficult to effectively image deep vasculature structures in human tissues like hand. Therefore, LSCI’s high-resolution capabilities are primarily limited to surface layers [13]. The FI was designed to selectively observe blood perfusion in the vasculature by combining the LSCI and SVI, providing more complementary information for vasculature disorders. While the FI was focused on the venous flow in this study, which is of particular interest in various medical diagnostic applications, it should be noted that this approach potentially reduces the visibility of capillary perfusion in the surrounding vasculature.

Dual-mode illumination, employing both a laser and LED, enables the simultaneous acquisition of LSCI, SVI, and FI without illumination mode switching. Consequently, mutual interference may occur, potentially causing noise artifacts in the SVI and resulting in relatively higher K’ values in the LSCI compared to single-mode illumination. In fact, LSCI resulted in differences in K’ values between single- and dual-mode illumination at the same RPM as shown in Figs. 5, 6, and 7, likely because the LED contributed to the laser intensity under dual-mode illumination. As shown in Fig. 3, single-mode illumination produced lower laser intensity and higher fluctuation of laser speckle, resulting in lower K’ (higher K) values. Dual-mode illumination produced higher intensity probably due to the contribution of LEDs and lower fluctuation of laser speckle due to lower laser intensity, resulting in in higher K’ (lower K) value. Such interference could lead to inaccuracy in obtaining clear vascular morphology and blood perfusion. However, these artifacts may be a trade off in FI due to the increased speed achieved by simultaneously implementing LSCI, SVI, and FI. The artifact due to dual-mode illumination may be solved by interleaving the light sources of laser and LED [9]. Images may be acquired and processed in real time, provided that the interleaving speed is sufficiently fast for the real-time processing of LSCI, SVI, and FI. When the light sources are interleaved, it is necessary to separately acquire and process LSCI and SVI. This will necessitate comprehensive modifications to both hardware and software, which should be addressed in future studies.

Under both illumination modes, the FI was obtained by combining the LSCI and SVI. The LSCI was obtained through a moving window average calculation, which is faster than obtaining the SVI through contrast-enhanced image processing. LSCI and SVI were implemented at 38.4 and 18.08 fps, respectively, resulting in a two-fold difference in speed that finally caused a lower image processing speed of 11.36 fps in FI. The image processing speed may be further enhanced by developing a deep learning algorithm [36] and using a higher-performance CPU and GPU in future studies [37, 38].

Real-time projection mapping of FI can be achieved under dual-mode illumination by simultaneously obtaining both LSCI and SVI, and then subsequently combining them. However, under single-mode illumination, real-time projection mapping may not be achieved with FI because the illumination should be selectively switched to acquire LSCI and SVI, requiring a user-dependent switching time. FIs under single-mode illumination may be more effective for quasi-static evaluation before and after treatment because it requires a longer image processing time. FIs under dual-mode lighting may be more effective for time-varying evaluations during treatment because it provides a relatively faster image processing speed; however, it could result in a lower image quality.

The DOPMS may be used for diagnosing various venous disorders, such as the following: (1) venous stenosis, a condition characterized by the narrowing of the blood vessels in the legs [39]. This can lead to blood clots and deep vein thrombosis, which are serious medical conditions that require prompt treatment [40]; (2) superficial venous thrombosis or phlebitis, a condition characterized by inflammation of the superficial veins in the legs [41–43]; and (3) chronic venous insufficiency, in which the veins in the legs are unable to effectively pump blood back to the heart, leading to a buildup of blood in the legs and the formation of varicose and spider veins [44, 45]. The DOPMS may also be used for diagnosing and monitoring the progression of venous ulcers, which are painful open sores that can occur on the legs [46].

## Conclusion

The DOPMS enables a comprehensive evaluation of both the morphology and blood flow of the vasculature directly on the IROI by projecting images. It may be a valuable biomedical optics imaging tool for diagnosing various venous disorders and for postoperative monitoring. Further studies should be conducted on various biomedical applications for the potential utilization of the DOPMS.
